# Diagnostic Value of Sonographic Features in Distinguishing Malignant Partially Cystic Thyroid Nodules: A Systematic Review and Meta-Analysis

**DOI:** 10.3389/fendo.2021.624409

**Published:** 2021-03-19

**Authors:** Xinlong Shi, Ruifeng Liu, Luying Gao, Yu Xia, Yuxin Jiang

**Affiliations:** Department of Ultrasound, Peking Union Medical College Hospital, Chinese Academy of Medical Science, Beijing, China

**Keywords:** meta-analysis, diagnostic values, sonographic features, partially cystic thyroid nodules, thyroid carcinoma

## Abstract

Ultrasonography (US) is one of the most important methods for the management of thyroid nodules, which can be classified as solid, partially cystic, or cystic by composition. The various Thyroid Imaging Reporting and Data System classifications pay more attention to solid nodules and have reported pertinent US features associated with malignancy. However, the likelihood of malignancy of partially cystic thyroid nodules (PCTNs) is 3.3–17.6%, and few studies have systematically discussed the value of US in differentiating such entities. Therefore, we deemed it necessary to perform a systematic evaluation of US features in recognizing malignant PCTNs. Our systematic review and meta-analysis aimed to assess the value of US features in predicting malignant PCTNs. We searched the PubMed/MEDLINE, Web of Science, and Cochrane Library databases to find studies that researched US features of PCTNs and that were published before June 2020. Review Manager 5.3 was used to summarize suspicious US features and calculate the sensitivity, specificity, and likelihood ratios. MetaDiSc 1.4 was used to estimate receiver operating characteristic curves and calculate areas under the curves (AUCs). Our review included eight studies with a total of 2,004 PCTNs. Seven features were considered to be associated with malignancy. High specificity (>0.9) was found in nodules with a taller-than-wide shape, those that were spiculated/microlobulated or with an ill-defined margin, those with microcalcification, and a non-smooth rim. Among US features, eccentric configuration, microcalcification, and marked or mild hypoechogenicity were more reliable in predicting malignancy (AUC: 0.9592, 0.8504, and 0.8092, respectively). After meta-analysis, we recommend combining PCTN US features including an eccentric internal solid portion, marked or mild hypoechogenicity, and presence of microcalcification to better identify malignant nodules. More studies are needed to explore and improve the diagnostic value of US in PCTNs.

## Introduction

Ultrasonography (US) is one of the most important methods for the management of thyroid nodules (TNs). In clinical practice, a nodule can be classified as solid, partially cystic, or cystic based on the internal cystic components (1). The various Thyroid Imaging Reporting and Data Systems (TI-RADS) classifications have paid more attention to solid nodules and have reported pertinent US features associated with malignancy (1–5). Several studies reported that nodules with microcalcification, hypoechogenicity (mild or marked), a taller-than-wide shape, or a spiculated/microlobulated margin are more likely to be carcinoma (6–9). However, the likelihood of malignancy of partially cystic thyroid nodules (PCTNs) is 3.3–17.6%, and few studies have systematically reported the US features associated with malignant PCTNs and discussed the value of US in differentiating such entities. As a matter of fact, malignant PCTNs can be easily missed due to their low prevalence (10–14). Therefore, we consider that more attention should be paid to the diagnosis of malignant PCTNs. Our systematic review and meta-analysis aimed to identify US risk factors indicative of malignant PCTNs and to assess the diagnostic performance of these features.

## Materials and Methods

### Search Strategy

This meta-analysis was referred to Perfected Reporting Items for Systematic Review and Meta-analysis guideline (15). We searched the PubMed/MEDLINE and Web of Science databases to obtain relevant literature for this review. In the PubMed/MEDLINE database, the following search terms were conducted: (partially cystic thyroid nodules [MeSH Major Topic]) AND (ultrasonograph* OR sonograph* OR ultrasound OR US [MeSH Major Topic]). The advanced search terms “TS=[(partially cystic thyroid nodules) AND (ultrasoundgraph* OR sonograph* OR ultrasound OR US)]” were used in the Web of Science database. We also checked the Cochrane Library with “partially cystic thyroid” AND “ultraso*.” We did not screen according to language. From a search up to June 2020, 56 articles (31 in Web of Science and 25 in PubMed) in total were identified. There were no relevant studies registered in the Cochrane Library. All articles were managed with NoteExpress V3.0 and duplicated studies were manually deleted.

### Inclusion and Exclusion Criteria

**Figure 1:**
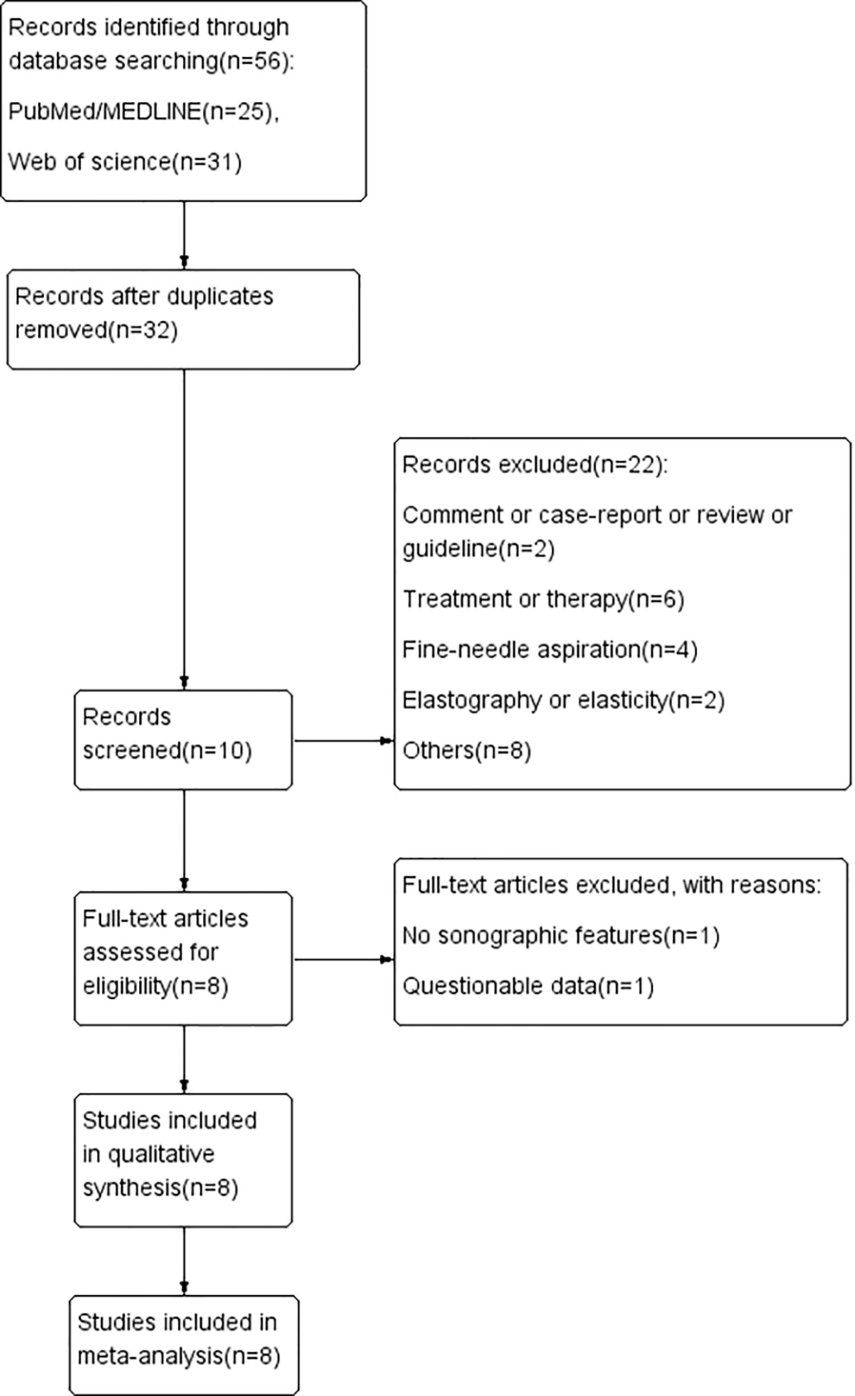
Flow chart of literature review process. Finally, a total of eight studies were included in our review.

After searching the databases and deleting duplicated articles, we tab retained 56 studies for further analysis. Subsequent selection was performed by screening the titles and abstracts of all retrieved records. Comments, case reports, conference abstracts, letters, or reviews were filtered. The last round of selection was to apply strict and distinct inclusion and exclusion criteria by reviewing the full texts. Articles that met the following criteria were included in this study: (1) study on the sonographic features of PCTNs; (2) histopathologic results used as a reference standard; (3) research results available for evaluating the diagnostic value of sonographic features in PCTNs; (4) retrospective or prospective study. The exclusion criteria were as follows: (1) studies on themes other than PCTNs; (2) diagnostic classification or no specific sonographic features about PCTNs; (3) insufficient or questionable data to finish a diagnostic 2-by-2 table; (4) improper deletion of studied cases. Finally, a total of eight studies (16–23) were retained according to the selection procedure in **Figure 1**.

### Data Extraction

Two radiologists (XS and RL) individually reviewed the selected literature and extracted the data for systematic review and meta-analysis. We collected the following information from the selected articles: basic characteristics (name of first author, year of publication, country of origin, study design, number of TNs, number of included PCTNs, and scanner), sonographic performance of PCTNs, and diagnostic index of US features. According to several studies (1–5), some US features were excluded, such as vascularity. We regarded ovoid, ovoid-to-round, flat and round, and regular and parallel nodules as being wider-than-tall (anteroposterior/transverse diameter [A/T] <1) and irregular-shaped nodules were classified as taller-than-wide (A/T ≥1). Any discrepant data were discussed by XS and RL and a specialist (YX) with over 20 years of experience to reach consensus.

### Quality Assessment

QUADAS-2, a recommended tool for diagnostic accuracy studies (24, 25), was used by two reviewers to evaluate the quality of the eight included studies. Another reviewer was consulted for evaluation when any disagreement occurred.

### Statistical Analysis

Our first step was to find the independent risk features for thyroid malignancy. An intervention review was created in Review Manager 5.3 to calculate odds ratios (ORs), 95% confidence intervals (CIs), and p-values and to evaluate the risk bias of the included articles. The I^2^ inconsistency index was calculated to determine whether heterogeneity existed. If I^2^ ≥ 50%, the heterogeneity could not be ignored, and therefore, a random-effects model would be recommended to replace the default model. Next, independent risk features were analyzed by MetaDiSc 1.4 software to evaluate the diagnostic performance for predicting malignancy. The relationship between sensitivity and 1-specificity determines whether a threshold effect exists. When p > 0.05, the threshold effect can be ignored when analyzing the source of heterogeneity. Without a threshold effect, we would directly calculate the pooled sensitivity (Se), specificity (Sp), positive and negative likelihood ratios (LR+ and LR−), diagnostic OR (DOR), and area under the curve (AUC). A hierarchical summary receiver operating characteristic curve (HSROC) should be used to calculate AUC when a threshold exists (26–29).

## Results

**Figure 2:**
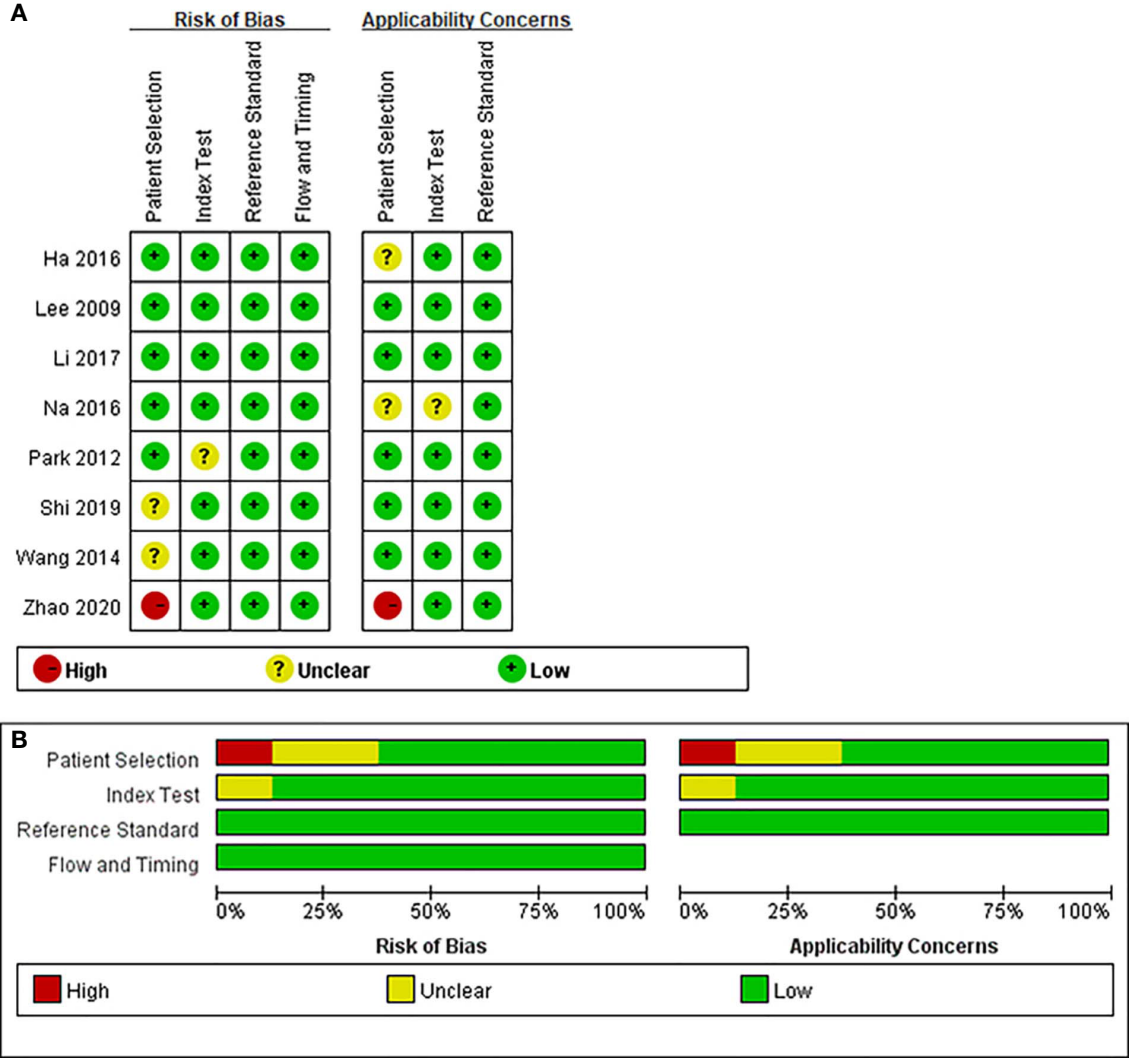
Outcome of QUADAS-2 for included studies. **(A)** Risk-of-bias summary. **(B)** Risk-of-bias graph. Symbols: (+), low risk of bias; (?), unclear risk of bias; (-), high risk of bias.

**Figure 3:**
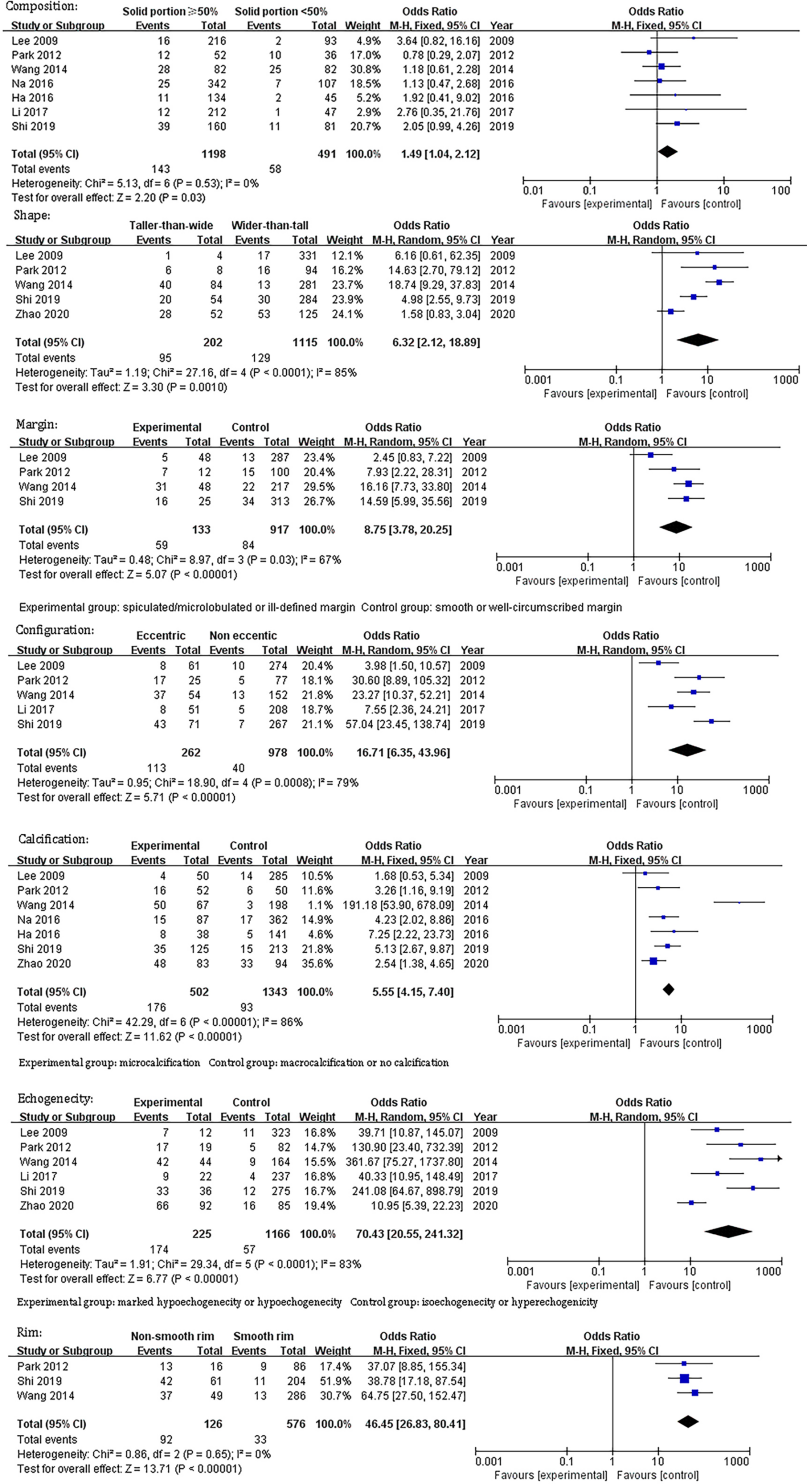
Odds ratio and its 95% confidence intervals of seven sonographic features of partially cystic thyroid nodules.

**Figure 4:**
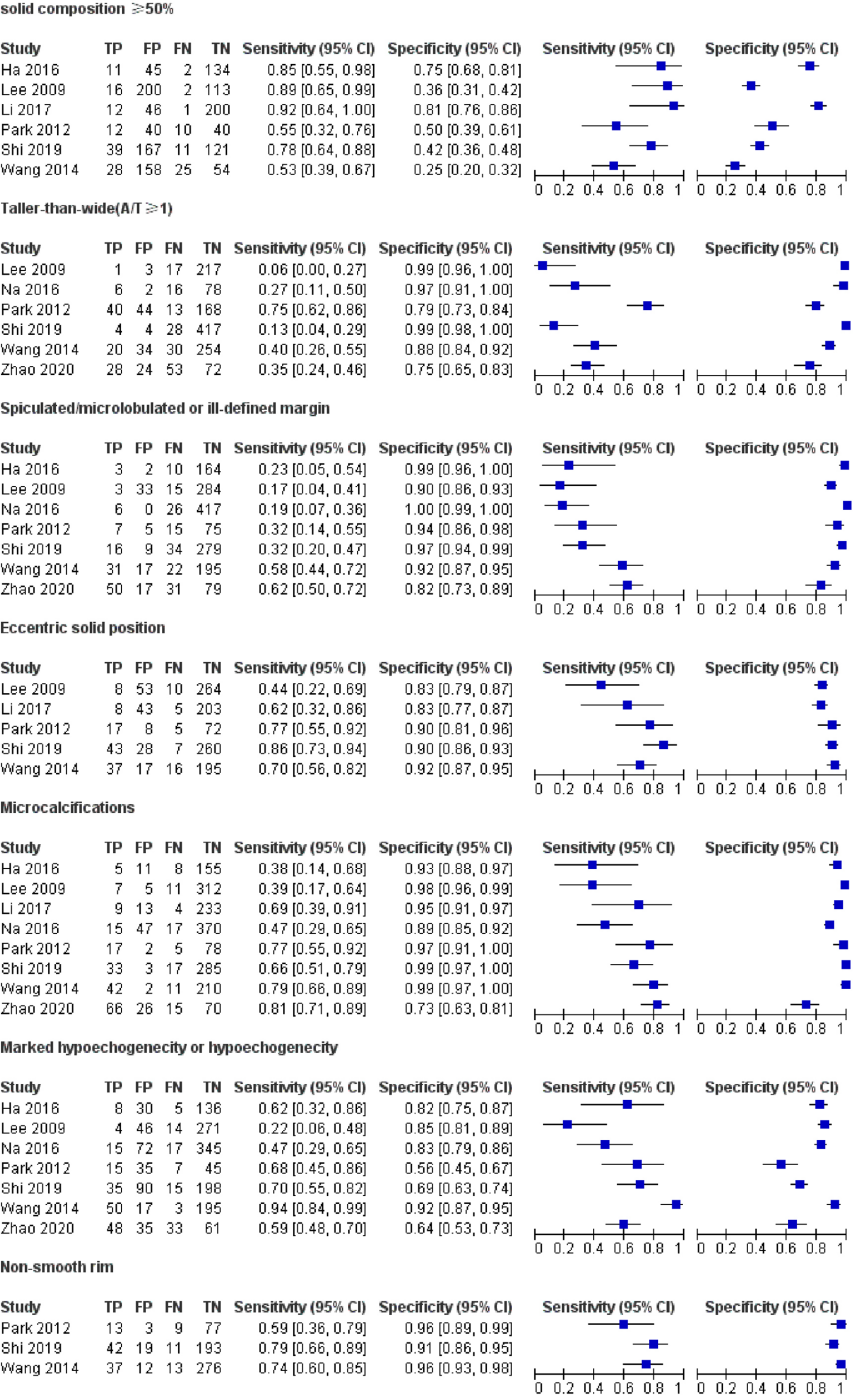
Forest plots of pooled sensitivity and specificity of US. Univariate analyses were performed for sensitivity and specificity, respectively. Except nodules with a solid portion ≥ 50%, the other six features revealed good specificity through a qualitative analysis.

**Figure 5:**
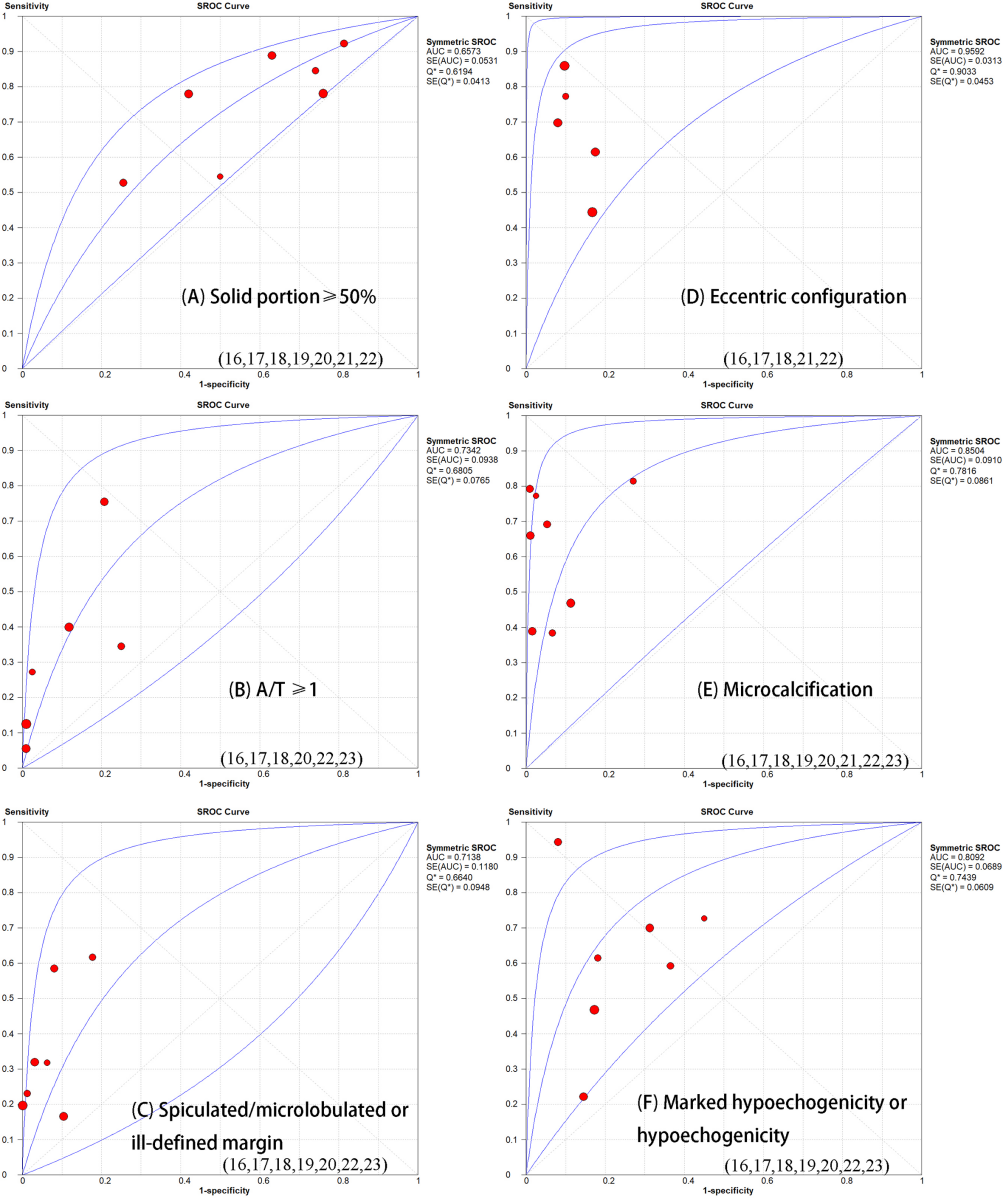
Summary receiver operator characteristic curve (SROC) with area under the ROC curve (AUC) of six sonographic features in diagnosing partially thyroid cancer. The size of each study is indicated by the size of the solid circles. PCTNs with an eccentric configuration are more prone to malignancy (AUC=0.09592).

**Table 1** demonstrates the basic information of the eight included studies. Half were performed in China (18, 21–23) and the other half were conducted in Korea (16, 17, 19, 20). **Figure 2** shows the outcomes of the QUADAS-2 questionnaire. All included studies had a low risk of bias and were of high quality. We noted that nodules were more prone to be malignant with internal solid content ≥50%, taller-than-wide shape, and when spiculated/microlobulated or with an ill-defined margin. In terms of internal solid content of a PCTN, eccentric configuration, a non-smooth rim, marked or mild hypoechogenicity, and microcalcification were also potential malignant features for PCTNs. More details are shown in **Figure 3**. The overall ORs of the seven suspicious features ranged from 1.49 to 70.43. The p-values of all features were <0.01 except for nodules with a solid portion ≥50% (p = 0.03). Then, we combined RevMan 5.3 and MetaDiSc 1.4 software to evaluate the diagnostic accuracy. **Figure 4** and **Figure 5** show the pooled Se and Sp of diagnostic performance in the eight included studies. Except nodules with a solid portion ≥50%, the other six features revealed good specificity through a qualitative analysis. Four features (spiculated/microlobulated or ill-defined margin, eccentric configuration, microcalcification, and marked or mild hypoechogenicity) showed no threshold effect in this meta-analysis (p = 0.337, 0.285, 0.955, 0.760, respectively). Hence, we could obtain pooled diagnostic statistics from these four features. We only calculated the AUC from the HSROC for US features with an identified threshold effect. The pooled Se, Sp, LR+, LR−, DOR, 95% CIs, and AUCs are displayed in **Table 2**. From this table, we discovered that three features, except a non-smooth rim, of only the internal solid portion were more likely to predict the malignancy of PCTNs compared with features of the entire nodule (all AUCs >0.8). The AUC of the solid portion ≥50%, taller-than-wide shape, and spiculated/microlobulated or ill-defined margin were 0.6573, 0.7342, and 0.7138, respectively. Metaregression was conducted in MetaDiSc 1.4 to explore the source of heterogeneity. The variables were TP+FN (TP, True-positive; FN, False-negative), country of region, study design, and numbers of scanner used. We added year of publication to the metaregression of presence of microcalcification. We found that whether the study was conducted in China or South Korea was the main source of heterogeneity in terms of the presence of microcalcification (p = 0.0482, **Table S1**), while no other covariates could explain heterogeneity. We did not assess publication bias because our review included only eight studies, and the Cochrane Handbook recommends at least 10 studies when evaluating publication bias.

**Table 1:** Basic characteristic of included studies.

First author	Year of publishing	Country of region	Study design	No. of TNs	No. of PCTNs	Rate of PCTNs (%)	Included PCTNs
Mi Jung Lee (16)	2009	South Korea	Prospective	1,056	392	37.1	335
Jang Mi Park (17)	2012	South Korea	Retrospective	NA	102	NA	102
Xiaoqing Wang (18)	2014	China	Retrospective	NA	265	NA	165
Eun Ju Ha (19)	2016	South Korea	Prospective	1,109	NA	NA	179
Dong Gyu Na (20)	2016	South Korea	Retrospective	2,000	449	22.5	449
Wenbo Li (21)	2017	China	Prospective	1,360	281	20.7	259
You Zhen Shi (22)	2019	China	Retrospective	NA	338	NA	338
Hai Na Zhao (23)	2020	China	Retrospective	NA	200	NA	177

NA, not available.

**Table 2:** Diagnostic performance of each malignant feature.

Features	Se (95% CI)	Sp (95% CI)	PLR (95% CI)	NLR (95% CI)	DOR (95% CI)	AUC
Solid portion ≥50%	0.71 (0.64–0.77)	0.39 (0.37–0.41)	/	/	/	0.6573
A/T ≥1	0.39 (0.33–0.45)	0.92 (0.91–0.93)	/	/	/	0.7342
Spiculated/microlobulated or ill-defined margin	0.43 (0.37–0.49)	0.95 (0.94–0.96)	6.24 (3.39–11.47)	0.68 (0.56–0.84)	10.35 (5.21–20.54)	0.7138
Eccentric configuration	0.72 (0.65–0.80)	0.87 (0.85–0.89)	5.67 (3.42–9.38)	0.34 (0.20–0.59)	17.22 (6.53–45.41)	0.9592
Microcalcification	0.69 (0.63–0.74)	0.94 (0.93–0.95)	13.97 (6.10–31.97)	0.39 (0.27–0.55)	38.76 (6.10–31.97)	0.8504
Marked hypoechogenicity/hypoechogenicity	0.65 (0.59–0.71)	0.79 (0.77–0.81)	2.70 (1.66–4.38)	0.48 (0.30–0.77)	5.97 (2.47–14.43)	0.8092
Non-smooth rim	0.74 (0.65–0.81)	0.94 (0.92–0.96)	/	/	/	0.5

Se, sensitivity; Sp, specificity; PLR, positive likelihood ratio; NLR, negative likelihood ratio; DOR, diagnostic odds ratio; AUC, area under the curve.

## Discussion

In our review, the incidence of malignant PCTNs varied from 5.0 to 45.8%. The diagnosis of malignant PCTNs is challenging, but worthy. It is of great importance to identify sonographic features that distinguish malignant PCTNs in clinical practice. Hence, we conducted this systematic review and meta-analysis to evaluate the value of US in predicting malignant PCTNs. After conducting an intervention review to determine independent risk factors for malignancy, we found PCTNs with seven US features had a higher risk of malignancy. Some of these features were in line with a previous meta-analysis regarding risky US features in all kinds of thyroid carcinoma (10). In our study, except non-smooth rim (AUC = 0.5), the AUCs of other six features were above 0.5. Notably, eccentric configuration, marked or mild hypoechogenicity, or presence of microcalcification of internal solid portion had relatively high accuracy (0.85, 0.77, 0.90, respectively) in predicting malignancy among PCTNs.

A taller-than-wide (TTW) shape, defined as an anteroposterior/transverse diameter (A/T) ratio >1, would not be reliably correlated with malignant PCTNs in our review (AUC = 0.7342). Likewise, Kim reported that a taller than wide shape did not contribute to an increased risk of malignant PCTNs. The reason may lie in the noted inter- and even intraobserver variability of taller-than-wide shape (30, 31). Hypoechogenicity showed fair diagnostic performance in our review (AUC = 0.8092). A previous study (32) that subdivided TNs based on their degree of hypoechogenicity also found that TNs with marked or moderate hypoehcogenicity had significantly higher malignant risks than mild hypoechogenicity (p < 0.001). This feature related closely with malignancy from the perspective of pathology. Kim stated that the pathogenesis of marked hypoechogenicity were associated with fibrotic regression following collapsed hemorrhagic component (31). The lack of follicular tissue arrangement may also lead to the hypoechogenicity of malignant PCTNs (33). Microcalcification of internal solid portion was significantly associated with malignancy as well (AUC = 0.8504). The degeneration of tumor cells and additional collagen produced by tumor cells could lead to psammoma bodies, a histopathological marker of microcalcification (34). They are common in any kind of papillary thyroid carcinoma regardless of the internal content. To some extent, these could explain why PCTNs with hypoechogenicity or microcalcification are prone to be malignant.

When compared to PCTNs with an eccentric configuration with a blunt angle, those with an eccentric configuration and an acute angle are more strongly associated with malignancy (p < 0.001) (18), which was also reported by Kim et al. (35). This phenomenon could be illustrated by the theory that malignant PCTNs usually develop from the wall of thyroid cysts, and the previous study has shown that the real tumor tissue is more likely to localize to the base of papillomatous lesions (36). A comment (37) reported that eccentric configuration harbors different meaning between nodules with a solid portion ≥50% and solid portion <50% (p = 0.001). Only in predominant solid nodules, an eccentric position of solid component is a significantly malignant feature. This could explain why we did not find “solid portion ≥50%” as being a high-risk factor by itself for predicting malignancy (AUC = 0.6573). Hence, we recommend integrating nodules with a solid portion ≥50% with other potential US features in future studies. And we should be alert when a PCTN presented with predominant solid and eccentric configuration simultaneously.

In addition to univariate analysis, some studies combined multiple US features to evaluate the diagnostic performance of US for PCTNs (16, 19, 20). However, because the combination of US features in these studies were different, it was impossible for us to evaluate the diagnostic accuracy of combined US features by meta-analysis. Lee et al. (16) found a high sensitivity and negative predictive value using combined US features to predict malignancy in PCTNs. Another two studies drew the same conclusion that PCTNs would have an intermediate risk of malignancy if they presented more than one suspicious US feature (38, 39). The risk of malignancy increased as more suspicious US features were detected. Although different TI-RADS were put forward to evaluate the thyroid nodule, the attention paid to PCTNs were relatively less. Therefore, we suggest that clinicians focus on the following features: eccentric configuration, presence of calcification, and marked or mild hypoechogenicity. Overall, US has the ability to diagnose malignant PCTNs if high-risk features are appropriately recognized and interpreted.

Several limitations exist in our review. Firstly, only a small number of studies were used for this research, which rendered subgroup analysis ineffective when analyzing heterogeneity. Secondly, all included studies were performed in Asia, and so there may be population and race bias. Some features are closely associated and can exist simultaneously in malignant nodules (40); however the inherent relationship between suspicious US features could not be explored and we failed to evaluate the diagnostic value of combined US features. Then, more detailed classification of specific US feature could bring new insight, but we failed to do such research: for instance, included studies (19, 20, 29) in our review did not divide the degree of hypoechogenicity when exploring associated factors for malignancy, which limited our advanced analysis. Further study could be conducted to find the relationship between degree of hypoechogenicity and malignancy. Moreover, pooled data concerning the overall diagnostic value of US for PCTNs is not available.

## Conclusion

Our review selected high-quality published studies to analyze the performance of US when diagnosing malignant PCTNs. After meta-analysis, we found that several US features were highly accurate when diagnosing malignant PCTNs. With the aim of improving the diagnostic accuracy of US, we suggest combining several US features of the internal solid portion of PCTNs. More studies are needed to explore and improve the diagnostic value of US in PCTN.

## Data Availability Statement

The original contributions presented in the study are included in the article/**Supplementary Material**. Further inquiries can be directed to the corresponding authors.

## Author Contributions

XS and RL contributed equally to this review. RL conducted the work of search and collection of literature and helped XS to write the first draft of the manuscript. XS did the work of meta-analysis. LG contributed to the discussion. YX conceived and designed this review. YX and YJ revised the manuscript. All authors contributed to the article and approved the submitted version.

## Funding

This study was supported by a grant from the Tibet Autonomous Region Science and Technology Project (XZ201901-GB-04) and the Tibet Autonomous Region Organization and Aiding Project [XZ2019ZR ZY05(Z)].

## Conflict of Interest

The authors declare that the research was conducted in the absence of any commercial or financial relationships that could be construed as a potential conflict of interest.
